# Single-Cell-Based High-Throughput Cultivation and Functional Characterization of Biosurfactant-Producing Bacteria from Soil and Oilfield-Produced Water

**DOI:** 10.3390/microorganisms10112216

**Published:** 2022-11-09

**Authors:** Ying Xu, Yali Jing, Yaqian Zhang, Qingjie Liu, Jianlong Xiu, Ke Zhang, Ninghong Jia, Minghui Zhou, Xinyu Zhou, Jia Huang, Yong Nie, Xiao-Lei Wu

**Affiliations:** 1 State Key Laboratory of Enhanced Oil Recovery, PetroChina Research Institute of Petroleum Exploration & Development, Beijing 100083, China; 2 College of Chemical Engineering and Environment, China University of Petroleum, Beijing 102249, China; 3 College of Engineering, Peking University, Beijing 100871, China; 4 Institute of Ocean Research, Peking University, Beijing 100871, China; 5 Institute of Ecology, Peking University, Beijing 100871, China

**Keywords:** biosurfactant, single-cell culture, biosurfactant-producing bacteria, isolation, response surface methodology

## Abstract

Biosurfactants are a group of surface-active compounds that can be produced by diverse microorganisms. They have been widely used in various industrial fields. Reducing production costs, improving efficiency, and collecting more diverse producing strains have become major challenges in the biosurfactant industry. These challenges could be overcome by screening for more diverse and efficient biosurfactant-producing strains. The conventional methods for the isolation and functional characterization of microorganisms are laborious and biased toward fast-growing or strongly competitive microorganisms. Here, we established a high-throughput approach of single-cell-based cultivation and functional characterization of biosurfactant-producing bacteria (SCCBB). This approach combines single-cell cultivation with the detection of optical distortions. Using this approach, we isolated 431 strains with biosurfactant production potential from petroleum-contaminated soil and oilfield-produced water. The surfactant production capabilities of the strains were subsequently validated using surface tension measurements, TLC, and CMC measurements. To investigate the industrial production potential, we optimized the production conditions of a representative glycolipids-producing strain, *Pseudomonas* sp. L01, using response surface methodology (RSM). Optimal conditions yielded a crude biosurfactant yield of 8.43 g/L in a flask. Our work provides a high-throughput approach to the isolation and screening of biosurfactant-producing bacteria, as well as other functional bacteria in a wide range of fields.

## 1. Introduction

Biosurfactants are a group of amphiphilic surface-active compounds mainly including glycolipids, lipopeptides, lipoproteins, lipopolysaccharides, and phospholipids, which can be produced by diverse microorganisms, including bacteria, yeast, and fungi [1,2,3,4,5]. Surfactants have great advantages over chemically synthesized surfactants, such as low toxicity, high stability, high efficacy, and high biodegradability. Biosurfactants have been widely applied in agriculture, industry, and bioremediation [6,7,8,9,10]. Furthermore, because some biosurfactants, such as surfactin, exhibit significant anti-viral and anti-inflammatory effects, they are also considered potential therapeutic agents against infection [11]. The significant applicability potential of biosurfactants has stimulated the global biosurfactant market. It is reported that the market value of commercially available biosurfactants is expected to reach US $2.6 billion by 2023, generating 540 kilotons of supply by 2024 [12]. Reducing production costs, improving efficiency, and collecting more diverse strains of producers have become major challenges in the biosurfactant industry.

Benefiting from efforts to isolate and screen biosurfactant-producing microorganisms, a large number of producer strains have been identified. Of these microbial strains, members of the species *Pseudomonas aeruginosa* and *Bacillus subtilis* are the most attractive for commercial production due to their superior ability to produce rhamnolipids and surfactin, respectively. Additionally, many hydrocarbon degraders produce biosurfactants during hydrocarbon metabolisms, such as *Rhodococcus*, *Dietzia*, *Acinetobacter*, and *Marinobiacter*, which are thought to have potential in bioremediation and microbial enhanced oil recovery (MEOR) [13,14,15]. While several strains have been used in commercial production, the need for new biosurfactant-producing strains is growing. For example, the most popular producer strain, *Pseudomonas aeruginosa*, is also a potentially pathogenic strain that could pose a challenge to biosecurity. Moreover, limited structural variation hinders the application of biosurfactants in more fields. These challenges could be overcome by screening new compounds from diverse biosurfactant producers.

Conventional methods for isolating and culturing microorganisms are laborious, time-consuming, and biased toward those fast-growing or strongly competitive microorganisms [16,17]. The microorganisms with low cell densities, low rates of growth, and resistance to cultivation might be “unculturable”. To overcome these limitations, several approaches have been developed. For example, the diffusion chamber could simulate the environmental conditions which ensure that the microorganisms in the chamber could obtain essential nutrients and growth factors from the environment [18]. Another method for isolating low-density and slow-growing microorganisms is capturing and culturing single microbial cells [19,20]. This avoids competition between microorganisms and allows for the cultivation of a large number of microbial cells at once, which has been used in high-throughput cultivation of microorganisms of interest.

Developing fast and high-throughput methods for the detection of surfactant activity is also a challenge for the efficient collection of biosurfactant-producing bacteria. The conventional methods include surface and/or interfacial tension measurement, axisymmetric drop shape analysis profile (ADSA-P), glass-slide test, and the oil spreading method [21]. However, the above methods are time-consuming, which makes them unsuitable for high-throughput screening of biosurfactant-producing strains. To overcome these limitations, several methods have been developed. The drop-collapsing test is a rapid and sensitive method for screening bacterial colonies that produce surfactants [22,23]. Drops of cell suspensions of surfactant-producing strains collapse and spread completely over an oil-coated surface. Moreover, this method is correlated with the ability of the cultures to reduce surface tension [21]. In addition, a quantitative microplate analysis was developed based on the effect of concave fluid surface on the image of a grid viewed through the wells of a microplate [24]. Biosurfactants in the water wet the walls of the wells in the microplate, creating a concave fluid surface like a diverging lens. As a result, the image of the grid below the well is distorted, which can be easily determined by eye or image processing software. Both methods can be used as primary high-throughput methods for detecting biosurfactant producers.

Here, we established a high-throughput culturomics approach of single-cell-based cultivation and functional characterization of biosurfactant-producing bacteria (SCCBB). This approach combines high-throughput single-cell culture with the detection of optical distortions caused by suspensions containing biosurfactants. We validated this approach by successfully isolating biosurfactant-producing bacterial strains from soil and oilfield-produced water. The biosurfactant production conditions of a representative strain were optimized using the response surface methodology (RSM) method to test potentials in industrial applications.

## 2. Materials and Methods

### 2.1. Sample Collection and Preparation

Two petroleum-contaminated soil samples (i.e., oil-soil-1and oil-soil-2) and an oilfield-produced water sample were collected in this work. Oil-soil-1 was an artificial petroleum-contaminated soil as described previously [25]. Oil-soil-2 was collected from Dagang oilfield (Dagang Oil Field Ltd., Tianjin, China), located in Tianjin, China, in March 2019. The soil samples were sifted through a 20-mesh brass sieve to remove large particles. One gram of the sieved soil sample was dispersed in 10 mL of phosphate-buffered saline (PBS), vortexed for one minute, and the soil suspension was obtained by filtration with gauze. The soil suspension was then homogenized by shaking with a crusher at 3000 rpm for five minutes to disperse microbial cells.

The oilfield-produced water was collected from the Ba51 fault block in the Baolige oilfield (Huabei Oilfield Ltd., Inner Mongolia, China) located in the central part of Inner Mongolia, China, in March 2019. The samples were transported to the laboratory in ice within 48 h. The produced water was then filtered twice with gauze and collected for further experiments.

### 2.2. Single-Cell Cultivation and Functional Identification

Four kinds of media were used for isolation, i.e., the LB medium (5.0 g/L yeast extract, 10.0 g/L tryptone, 10.0 g/L NaCl; pH 7.0), TYG medium (1.0 g/L peptone, 1.0 g/L yeast extract, 6.34 g/L KCl, 0.5 g/L glucose, 1.2 g/L NaCl, 0.25 g/L MgSO_4_∙7H_2_O, 0.13 g/L K_2_HPO_4_, 0.22 g/L CaCl_2_∙2H_2_O, 0.17 g/L K_2_SO_4_; pH 7.0), TSB medium (17.0 g/L tryptone, 3.0 g/L soya peptone, 2.5 g/L K_2_HPO_4_, 2.5 g/L glucose; pH 7.0), and mineral medium with oilfield-produced water (17.907 g/L Na_2_HPO_4_∙12H_2_O, 7.8 g/L NaH_2_PO_4_∙2H_2_O, 5.0 g/L (NH_4_)_2_SO_4_, 5.0 g/L KCl, 0.2 g/L MgSO_4_∙7H_2_O, 0,05 g/L CaCl_2_∙2H_2_O, 10 mL trace element solution SL-4 (Coolaber, China), dissolved in 1 L of oilfield-produced water; pH 7.0).

To determine the dilution level for single-cell inoculation in the wells of a microplate, we assume that bacterial cells are distributed into different wells of a microplate following a Poisson distribution. Thus, the probability that a well receives a different number of cells can be calculated based on Equation (1).

(1) $$P\left(X=k\right)=\frac{\lambda^{k}}{k!}e^{-\lambda }$$

where P is the probability, k is the expected number of cells in a well, and λ is the ratio of the total number of cells to the number of wells receiving cells.

The total number of culturable cells in the sample was first calculated using the traditional plate counting method. The optimal dilution level can then be determined by a Poisson distribution, where most wells are expected to receive either one or two cells. For example, the cell suspension should be serially diluted to the theoretical cell density of 24 CFU/mL for cultivation in a 384-well plate (60 μL of sample in each well). At this dilution level, approximately 24.3% of the wells in a 384-well plate are expected to be blank. The actual dilution level should then be modified based on the initial culture in a 384-well plate, where 24% to 30% of the cells were clear after 7 days of cultivation.

The surfactant activity of each isolate was determined using a microplate assay [24]. The OD_600_ of the culture in the microplate was detected using a microplate reader to identify wells with successful culture. Then, 10 μL of supernatant of each well with turbid cultures was added to 90 μL of pure water in a 96-microwell plate. Distilled water was used as a negative control. The plate was then viewed with a sheet of grid-line paper underneath. Wells that showed optical distortions of the grid were thought to have surfactant activity and were selected for further analysis.

### 2.3. Phylogenetic Analysis

DNA was extracted from each colony on LB agar plates using the DNA isolation kit (Corning, Corning, NY, USA). The bacterial 16S rRNA gene was amplified by polymerase chain reaction (PCR) using universal primers 8F (5′-AGAGTTTGATCCTGGCTCAG-3′) and 1492R (5′-GGTTACCTTGTTACGACTT-3′). The PCR process was performed using 2 × Taq Master Mix (Vazyme, Nanjing, China) with a three-step PCR program (initial denaturation step at 95 °C for 5 min, followed by 30 cycles at 95 °C for 30 s, 55 °C for 30 s, and 72 °C for 1 min 30 s, followed by a final step at 72 °C for 10 min). The bacterial 16S rDNA sequences obtained were then aligned with known 16S rDNA sequences in EzBioCloud (https://www.ezbiocloud.net/, accessed on 25 January 2021). A phylogenetic tree was constructed using the neighbor-joining method in MEGA X software [26], selecting the Kimura two-parameter model. The evolutionary history was inferred using the neighbor-joining method [27]. The percentage of replicate trees in which the associated taxa clustered together in the bootstrap test (1000 replicates) are shown next to the branches. The evolutionary distances were computed using the maximum composite likelihood method [28] and are in the units of the number of base substitutions per site. All ambiguous positions were removed for each sequence pair (pairwise deletion option).

### 2.4. Surface Tension Measurement

The strain was first grown for 24 h at 30 °C in LB liquid medium by shaking at 220 rpm. The cells were then washed twice with PBS to make an inoculum. The inoculum was then transferred into the fresh LB medium containing 1/10 peptone with initial OD_600_ 0.1 and grown at 30 °C by shaking at 220 rpm. After cultivation, the cells were removed from the fermented broth by centrifugation at 8000 rpm for 10 min and the supernatants of each strain were harvested. Surface tension was measured using a video optical contact angle measuring device (Theta Flex, Biolin, Espoo, Finland) based on surface contact angle [29]. Each sample was measured three times. LB medium with 1/10 peptone was used as a blank control.

### 2.5. Biosurfactant Characterization (Crude Biosurfactant Extraction and TLC)

The cells were cultured in an LB medium containing 1/10 of the peptone described above. After culture, each fermented broth was centrifuged at 5000 rpm for 10 min. The resulting supernatant was acidified to pH 2 with 6 M HCl and placed at 4 °C overnight. The crude biosurfactant extract was obtained after centrifugation at 8000 rpm for 15 min at 4 °C to remove the supernatant and dry at 90 °C to constant weight. The detection of the biosurfactant extraction was performed by thin-layer chromatography (TLC). The solvent system used was chloroform:methanol:H_2_O (65:15:2, *v*/*v*/*v*). The resulting spots on the TLC were visualized using the spray reagent ninhydrin-acetone (0.5%, *m*/*v*) for lipopeptide and anthrone-concentrated sulfuric acid (0.2%, *m*/*v*) for glycolipid [14].

### 2.6. Biosurfactant Production Optimization Using Response Surface Methodology

For biosurfactant production optimization, the effects of different carbon sources (i.e., glucose, glycerol, and soybean oil) and nitrogen sources (i.e., yeast extract, peptone, urea, NaNO_3_, and (NH_4_)_2_SO_4_) on the yield of glycolipids were detected. The minimal medium (pH 7.0) consisted of 10 g/L Na_2_HPO_4_∙12H_2_O, 10 g/L KH_2_PO_4_, and 0.5 g/L MgSO_4_, which was supplemented with different carbon sources and nitrogen sources to evaluate the effects of carbon and nitrogen sources. After the carbon source and nitrogen source were determined, the medium was optimized using response surface methodology (RSM). The RSM experiment was designed using the Design Expert software (V12, State-Ease, Minneapolis, MN, USA). All experiments were performed in an Erlenmeyer flask with 50 mL of media.

### 2.7. Statistical Analysis

All experiments in this work were performed at least in triplicate. All data in the experiment were expressed as mean ± standard deviation (SD). The data were statistically analyzed using a two-tailed unpaired Student’s t-test, where a *p*-value of 0.05 or less is considered statistically significant.

## 3. Results and Discussion

### 3.1. Workflow of the SCCBB Approach

**Figure 1:**
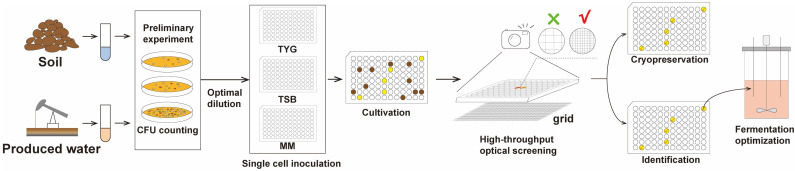
Schematic workflow of high-throughput single-cell-based cultivation and functional characterization of biosurfactant-producing bacteria.

Conventional methods for isolating and identifying biosurfactant-producing bacteria are inefficient and time-consuming, often relying on the isolation of colonies in a petri dish and functional characterization of scaled-up cultures in a flask. Moreover, cultivation based on colony selection suffers from severe cultivation bias due to the different growth fitness of various species. To overcome this problem, we established a high-throughput culturomics approach of single-cell-based cultivation and functional characterization of biosurfactant-producing bacteria (SCCBB). The SCCBB approach combined single-cell cultivation and high-throughput screening based on the detection of the optical distortion through the supernatant containing surfactants (Figure 1). In this approach, we inoculated the single bacterial cell from samples in a 96-well or 384-well plate with different media using limiting dilution. The results showed that ~74% of the wells with visible bacterial growth were pure cultures, ~24.6% contained two strains, and ~1% contained more than two strains. The results showed that such single-cell cultures can isolate strains in liquid culture, allowing for further identification of supernatants without the need for additional colony pick-and-pass cultures.

**Figure 2:**
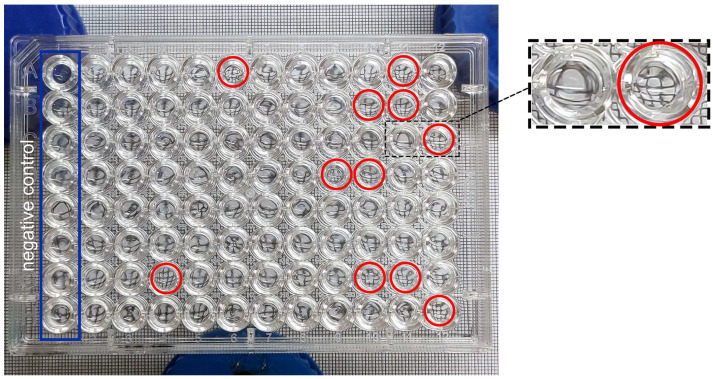
High-throughput screening of the cultures with surfactant activities by detecting the optical distortion of the grid. The red circles indicated the wells with surfactant activities.

Traditional methods to detect surfactants, such as surface tension measurements and oil dispersal assays, are time-consuming and limit high-throughput screening of biosurfactant-producing bacteria. Here, we screened the surfactant activity from the cultures using a microplate method [24]. The culture was transferred directly to a 96-well plate, which was placed on a grid of paper. The solution containing biosurfactants could form a concave surface and magnify the view of the grid (Figure 2). By taking a photograph of the plate, the wells containing biosurfactants can be easily identified. In this way, the method is time-saving. Primary isolation and screening can be completed within a week after the dilution level is determined.

### 3.2. Single-Cell-Based Inoculation Reduced the Cultivation Bias

**Figure 3:**
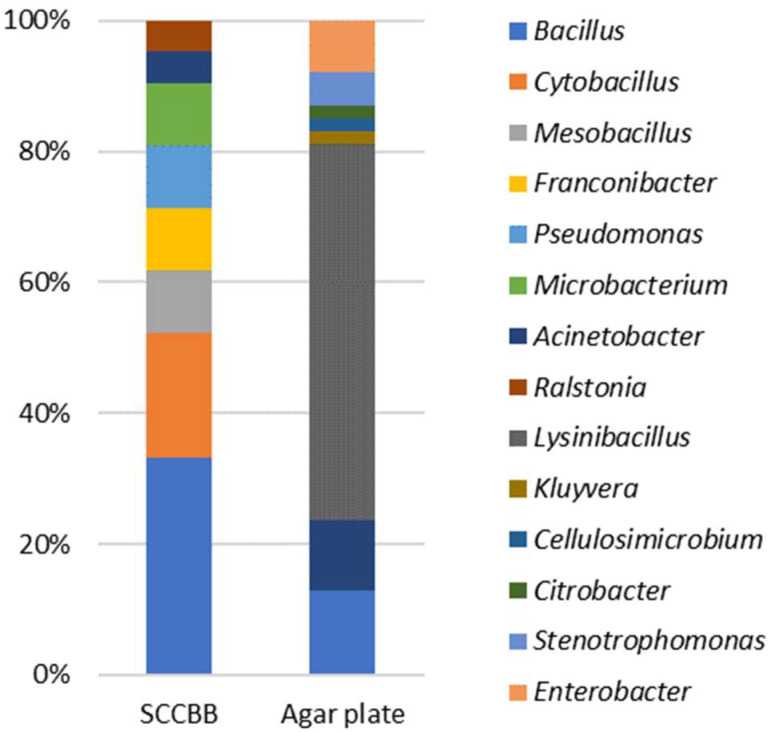
Taxonomic comparison of strains isolated using SCCBB and agar plate methods.

To assess the cultivation bias of the SCCBB method, we isolated twenty-one potential biosurfactant-producing bacterial strains from a petroleum-contaminated soil sample using this method. These strains belonged to eight genera (Figure 3). As a comparison, strains were also isolated from the same sample using a traditional agar plate [25]. Sixty-two colonies were picked from the agar plate and could be assigned to eight genera (Figure 3). We compared the taxonomic distribution of the isolates from both two methods. The results indicated that isolates from SCCBB methods showed higher evenness than those from the agar plate. About 58% of the total number of strains from the agar plate belonged to the genus *Lysinibacillus*. Moreover, using the agar plate method, we failed to isolate strains belonging to the genus *Pseudomonas*, which are well-known biosurfactant-producing bacteria. It has been reported that the genus *Lysinibacillus* is fast-growing, stress-tolerant, and can induce the abnormal biofilms of other species by signaling molecules [30,31,32]. Consequently, strains with higher growth fitness, such as *Lysinibacillus*, tend to be over-represented in culture conditions, suggesting that differences in growth fitness can lead to a cultivation bias when using the agar plate method.

### 3.3. Isolation of Biosurfactant-Producing Bacterial Strains from Soil and Produced Water Samples

**Figure 4:**
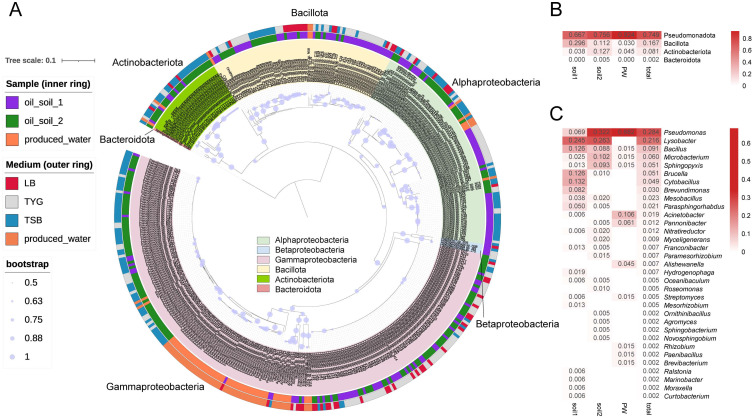
Taxonomic distribution of isolates from petroleum-contaminated soil and oilfield-produced water samples using the SCCBB approach. (**A**) Phylogenetic tree based on the 16S rRNA sequences of the isolates. (**B**) The taxonomic compositions of isolates from different samples at the phylum level. The numbers in the graduated color blocks represent the relative abundances of the taxa in the sample. (**C**) The taxonomic compositions of isolates from different samples at the genus level. The numbers in the graduated color blocks represent the relative abundances of the taxa in the sample. (**B**,**C**), soil1, oil-soil-1; soil2, oil-soil-2; PW, oilfield produced water.

Three samples, including two petroleum-contaminated soil samples and one oil-produced water sample, were selected for the isolation of biosurfactant-producing bacteria using different media using the SCCBB method. After 7 days of single-cell cultivation, all wells were screened by optical distortion. A total of 431 isolates with biosurfactant-producing potentials were obtained (Figure 4A). Based on the 16S rRNA gene analysis, most of the isolates (380 isolates) showed relatively high sequence similarities (>99%) with validly published strains, 17 isolates shared 98.65–98.99% similarities with validly published strains, and 34 isolates shared relatively low 16S rRNA gene sequence similarities (<98.65%) with validly published strains, which was proposed as the threshold for species-level identification [33]. These 431 isolates could be assigned to four phyla (i.e., Proteobacteriota, Bacillota, Actinobacteriota, and Bacteroidota) (Figure 4A) and 33 genera (Figure 4C) dominated by *Pseudomonas* (28.8% of the total isolates), *Lysobacter* (21.6%), *Bacillus* (7.9%), *Microbacterium* (5.8%), *Sphingopyxis* (5.3%), and *Brucella* (5.1%). Of these genera, *Pseudomonas* strains isolated from all samples, accounted for nearly 70% of the abundance in oilfield-produced water. This result is consistent with the microbial compositions of oil reservoir microbiomes, which showed that *Pseudomonas* was the most abundant genus in the oilfield-produced water samples [34]. In addition, high abundances of *Acinetobacter* and *Pannonibacter* have also been isolated from the oilfield-produced water sample. *Lysobacter*, in contrast, has only been isolated from soil samples with relatively high abundances.

### 3.4. Functional Verification of the Isolates

**Figure 5:**
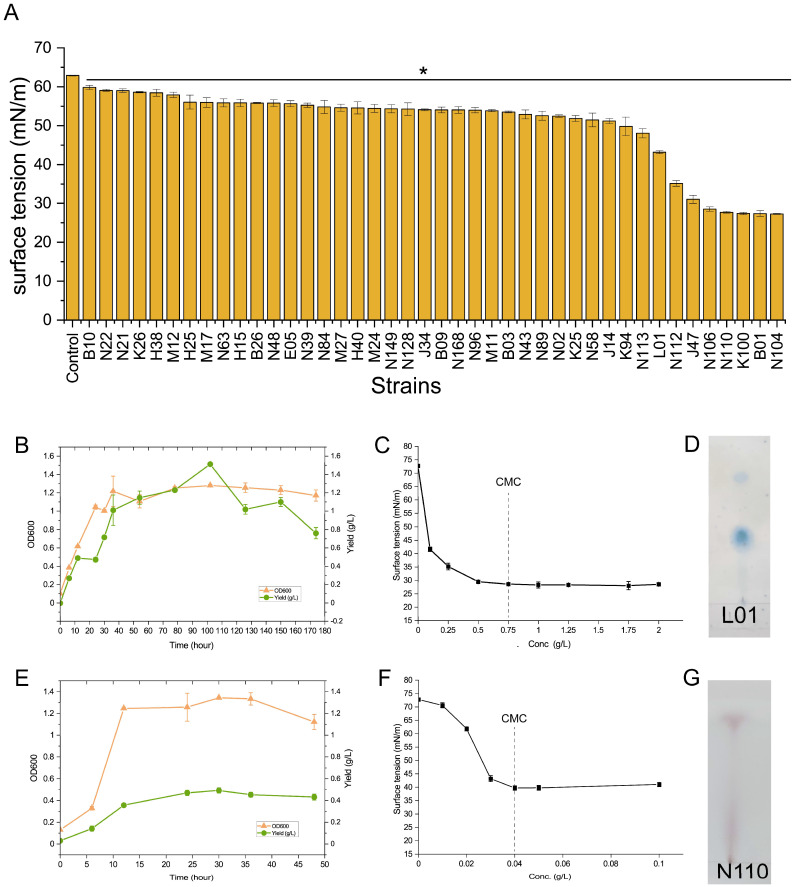
Functional verification of the representative strains isolated using the SCCBB approach. (**A**) The surface tension of the culture suspensions. * *p* < 0.05 relative to the culture medium by Student’s *t*-test. (**B**) Growth and yields of crude biosurfactants produced by *Pseudomonas* sp. L01. (**C**) Surface activities of the crude biosurfactants produced by L01. (**D**) Thin-layer chromatography (TLC) analysis of the crude biosurfactants produced by L01. The band indicates the presence of glycolipids with the anthrone-sulfuric acid reagent. (**E**) Growth and yields of crude biosurfactant produced by *Bacillus* sp. N110. (**F**) Surface activities of the crude biosurfactants produced by N110. (**G**) TLC analysis of the crude biosurfactants produced by N110. The band indicates the presence of lipopeptides with ninhydrin solution in acetone.

Based on the 16S rRNA gene sequences, all 431 isolates can be assigned to 99 phylotypes (i.e., 16S rRNA gene sequences shared 100% identity in a phylotype). We randomly selected one candidate strain for each phylotype for further analysis. To assess the capacity of the isolates to produce biosurfactants, we measured the surface tension of the culture supernatant for each candidate strain. The results showed that 42 strains significantly reduced the surface tension of the culture medium (Figure 5A), which suggested that these strains produced surface active agents. Five strains were able to reduce the surface tension of the medium to less than 30 mN/m, all belonging to *Bacillus*. In addition, the strain L01 could reduce the surface tension of the medium to 43.16 ± 0.14 mN/m, representing the most active strain in the genus of *Pseudomonas*.

To verify whether the decrease in surface tension was caused by the biosurfactants, we isolated and identified the surface active products from two representative strains with high surface activities (i.e., *Pseudomonas* sp. L01 and *Bacillus* sp. N110). Note that the cultivation conditions and growth rate for the two selected strains are different, so the periods for measuring growth and biosurfactant production are different for the two strains. We cultured *Pseudomonas* sp. L01 in a minimal medium with 2% (*w*/*v*) glucose as the sole carbon source and monitored the acid-precipitated crude biosurfactants at different time points. The results showed that the growth of L01 entered the stational phase after 24-h culture when the crude biosurfactant started to accumulate quickly (Figure 5B). The yield of crude biosurfactants reached the highest level of 1.51 ± 0.01 g/L at 102 h after inoculation. The activity of the crude biosurfactants was further determined by measuring the critical micelle concentration (CMC) [35]. The surface tension of pure water decreased from 72.77 ± 0.27 mN/m to the minimum of 28.6 ± 0.26 mN/m when 0.75 mg/mL of crude biosurfactants were added (Figure 5C). Thin-layer chromatography analysis showed that glycolipid dominated in the crude biosurfactants (Figure 5D). Using a similar approach, we also determined the biosurfactants produced by *Bacillus* sp. N110. The growth of N110 reached its stational phase 12 h after culture and the highest yield of crude biosurfactants was developed 30 h after culture (Figure 5E). The CMC value was 0.04 mg/mL for the crude biosurfactants of N110, at which the minimum surface tension was 39.71 ± 0.82 mN/m (Figure 5F). TLC analysis showed that lipopeptides dominated in the crude biosurfactants of the strain N110 (Figure 5G). It has been reported that the typical biosurfactants produced by *Pseudomonas* and *Bacillus* are glycolipids (e.g., rhamnolipid) and lipopeptides (e.g., surfactin), respectively [36]. Our results showed that the activity of representative strains was consistent with previous reports, confirming the feasibility of the SCCBB approach for isolating biosurfactant-producing bacteria. Moreover, this approach is function-oriented and time-efficient compared to conventional methods. This can be done within a week after the dilution level has been determined. Considering that a single researcher can handle 5 to 10 384-well microplates, we estimate that more than 200 potential biosurfactant-producing strains can be isolated per day by a single researcher.

### 3.5. Optimization of the Glycolipids Yield of Pseudomonas sp. L01 Using Response Surface Methodology (RSM)

To further investigate the potential of the strains for the industrial production of biosurfactants, we optimized the biosurfactants yield of *Pseudomonas* sp. L01 using the response surface methodology (RSM). We first evaluated the effects of different carbon sources (i.e., glucose, glycerol, and soybean oil) and nitrogen sources (i.e., yeast extract, peptone, urea, NaNO_3_, and (NH_4_)_2_SO_4_) on the yield of glycolipids. The yields of crude biosurfactants were determined in cultures with different concentrations of each carbon or nitrogen source at different time points. The results showed that yields of the crude biosurfactants in the carbon source test (2.5 g/L (NH_4_)_2_SO_4_) were used as the nitrogen source) reached the highest level when 30 g/L of glucose or more was used. The maximum yield in the nitrogen source test (30 g/L glucose was used as the carbon source) was 6.55 ± 0.53 g/L when 3.0 g/L NaNO_3_ was used.

The Plackett–Burman (PB) design was used to evaluate the most significant variables in the production of biosurfactants for strain L01. In addition to the carbon and nitrogen sources, we include the PO_4_^3−^ concentration, pH, and culture time as variables. The two-level values of these variables and the analysis of variance are shown in Table 1. We found that the concentration of NaNO_3_ and the time of culture were significant variables in the production of biosurfactants, with positive effects on the yield of biosurfactants.

According to the PB results, we selected the concentration of NaNO_3_, the cultivation time, and the concentration of PO_4_^3−^ as the factors for the further steepest ascent experiment (Table 2). The maximum yield was observed in the fourth step of the steepest ascent experiment and the levels of each factor were used as the central point for Box Behnken (BB) experiment (Table 3). The results were fitted to a second-order polynomial equation. The yield of biosurfactant (Y) could be predicted using the fitted quadratic regression equation below:

(2) $$Y=8.21-0.4175B-0.125C+0.03E+0.0325\mathrm{BC}-0.0075\mathrm{BE}-0.0075\mathrm{CE}-1.15B^{2}-0.18C^{2}$$

where, the coded values A, B, C, D, and E show the model terms glucose concentration, NaNO_3_ concentration, PO_4_^3−^ concentration, pH, and culture time. The *p*-value of the model was 0.0012 and the determination coefficient R^2^ was 0.9477, indicating that the experimental data fitted the model well.

**Figure 6:**
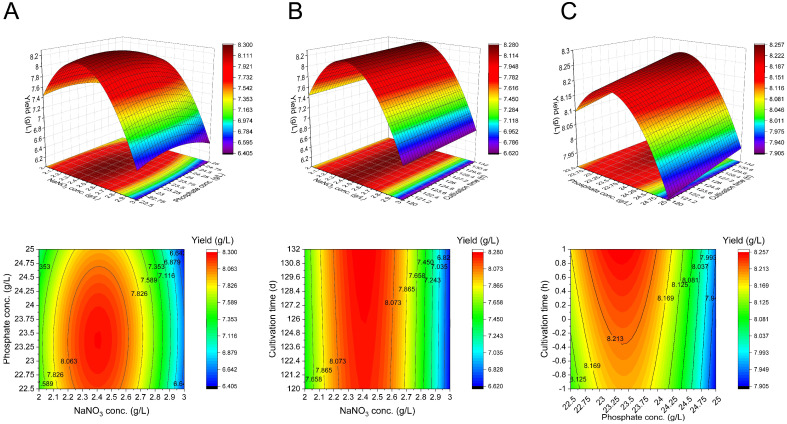
Three-dimensional surface and contour plots showing the interactions of factors on the biosurfactants yield. (**A**) Interactions between NaNO_3_ concentration and PO_4_^3−^ concentration (**B**) Interactions between NaNO_3_ concentration and cultivation time. (**C**) Interactions between PO_4_^3−^ concentration and cultivation time.

The combined effects of NaNO_3_ concentration vs. PO_4_^3−^ concentration, NaNO_3_ concentration vs. cultivation time, and PO_4_^3−^ concentration vs. cultivation time were determined by the three-dimensional surface and contour plots (Figure 6). According to the equation, the optimal values of NaNO_3_ concentration, cultivation time, and PO_4_^3−^ concentration were 2.4 g/L, 132 h, and 23.3 g/L, respectively. The predicted yield of the biosurfactants was 8.30 g/L. To validate the prediction, we perform the experiments using the optimal conditions. The yield of the biosurfactants under the predicted conditions was 8.43 ± 0.04 g/L, which was closer to the prediction and much higher than the yield (6.55 g/L) before RSM optimization.

## 4. Conclusions

In this work, we developed a high-throughput and function-oriented culturomics method for isolating and screening the biosurfactant-producing bacteria, which combined single-cell culture and optical distortion detection. Using this method, we isolated 431 strains with biosurfactant production potential from petroleum-contaminated soil and oilfield produced water. Since the microplate is well-compatible with automated instruments such as liquid handling systems, the throughput will be even higher. It can also be embedded in an integrated automated laboratory. Thus, our work provides an automatable solution for high-throughput isolation and screening of biosurfactant-producing bacteria, which also shed light on efficiently screening functional bacteria in other fields.

## Figures and Tables

**Table 1:** Plackett-Burman (PB) design for screening significant variables.

Variables	Low Level (−1)	High Level (+1)	Effect	*T*-Value	*p*-Value
glucose (g/L)	20	30	0.1120	1.19	0.281
NaNO_3_ (g/L)	1	3	1.0537	11.15	0.000
PO_4_^3−^ (g/L)	20	25	0.1637	1.73	0.134
pH	6.8	7.2	−0.1480	−1.57	0.168
time (h)	108	132	0.2520	2.67	0.037

**Table 2:** Steepest ascent experiment design and response values.

Trials	NaNO_3_ (g/L)	PO_4_^3−^ (g/L)	Time (h)	Yields (g/L)
1	1.0	20	108	3.55
2	1.5	21.25	114	6.09
3	2.0	22.5	120	6.97
4	2.5	23.75	126	8.23
5	3.0	25	132	6.06

**Table 3:** Analysis of variance (ANOVA) for regression model of Box Behnken experiment.

Source	Sum of Squares	df	Mean Square	*F*-Value	*p*-Value
Model	4.58	8	0.5725	114.11	0.0012 *
B (NaNO_3_)	1.39	1	1.39	277.96	0.0005 *
C (PO_4_^3−^)	0.0841	1	0.0841	16.75	0.0264 *
E (time)	0.0072	1	0.0072	1.44	0.3169
BC	0.0042	1	0.0042	0.8422	0.4264
BE	0.0002	1	0.0002	0.0449	0.8459
CE	0.0002	1	0.0002	0.0449	0.8459
B^2^	2.67	1	2.67	531.84	0.0002 *
C^2^	0.0648	1	0.0648	12.92	0.0369 *
E^2^	0.0000	0			
Residual	0.0150	3	0.0050		
Cor Total	4.59	11			

* *p* < 0.05.

## Data Availability

Not applicable.
